# Validation and Extrapolation of a Multimodal Infection Prevention and Control Intervention on Carbapenem-Resistant *Klebsiella pneumoniae* in an Epidemic Region: A Historical Control Quasi-Experimental Study

**DOI:** 10.3389/fmed.2021.692813

**Published:** 2021-07-07

**Authors:** Yunqi Dai, Tianjiao Meng, Xiaoli Wang, Bin Tang, Feng Wang, Ying Du, Yuzhen Qiu, Jialin Liu, Ruoming Tan, Hongping Qu

**Affiliations:** Department of Critical Care Medicine, Ruijin Hospital, Shanghai Jiaotong University School of Medicine, Shanghai, China

**Keywords:** infection prevention and control, carbapenem-resistant *Klebsiella pneumoniae*, intensive care unit, active surveillance, pre-emptive isolation, incidence

## Abstract

**Objective:** To verify the effects of comprehensive infection prevention and control (IPC) interventions for the prevention of the cross-transmission of carbapenem-resistant *Klebsiella pneumoniae* (CRKP) within intensive care units (ICUs) in an epidemic region.

**Methods:** A historical control, quasi-experimental design was performed. The study was conducted between January 2017 and December 2019, following the implementation of a multimodal IPC bundle. The baseline period was established from January 2013 to June 2013, when only basic IPC measures were applied.

**Results:** A total of 748 patients were enrolled during the entire study. The incidence of ICU-acquired CRKP colonization/infection was 1.16 per 1,000 patient-days during the intervention period, compared with 10.19 per 1,000 patient-days during the baseline period (*p* = 0.002). The slope of the monthly incidence of CRKP at admission showed an increasing trend (*p* = 0.03). The incidence of ICU-acquired catheter-related bloodstream infections caused by CRKP decreased from 2.54 to 0.96 per 1,000 central-line-days (*p* = 0.08). Compliance with contact precautions and terminal room disinfection improved during the intervention period. All environmental surface culture samples acquired after terminal room disinfection were negative for CRKP.

**Conclusion:** Our findings suggest that in epidemic settings, multimodal IPC intervention strategies and consistent monitoring of compliance, may limit the spread of CRKP in ICUs.

## Introduction

At the turn of the century, the surge in antimicrobial resistance (AMR) became a global public health threat. Current estimations suggest that AMR might cause as many as 10 million deaths per year by 2050, resulting in colossal morbidity and economic costs (1). *Klebsiella pneumoniae* (KP) can cause numerous infections in hospitals, long-term care facilities, and communities worldwide. Carbapenem-resistant *Klebsiella pneumoniae* (CRKP) is defined as any KP strain that is resistant to carbapenems or produces enzymes that hydrolyze carbapenems. The 2019 World Health Organization (WHO) global priority pathogens list for the research and development of new antibiotics ranks carbapenem-resistant *Enterobacteriaceae* (CRE) within the critical priority category.

Patients in intensive care units (ICUs) are at high risk for acquiring CRKP due to previous antibiotics exposure (carbapenem, tigecycline, or β-lactam/β-lactamases inhibitor), invasive procedures, and surgical operations (2). The incidence of CRKP in ICUs has been reported at 13.8–20.8% (2–4). CRKP could be transmitted to high-risk patients through contacts among healthcare workers, patients, shared medical equipment, and the surrounding environment, resulting in CRKP colonization (5). Infections caused by CRKP are challenging to treat because CRKP is resistant to a wide range of antibiotics, including carbapenems and colistin, which are last-resort drugs used in clinical practice (6). In multilevel analysis, ICU-acquired infection was independently associated with a higher risk of mortality compared with community-acquired infection (odds ratio: 1.32 [95% confidence interval (CI): 1.10–1.60]; *p* = 0.003] (7). Thus, proper and effective infection prevention and control (IPC) interventions are urgently necessary to reduce the incidence of CRKP acquisition in ICU settings (8).

National and global institutions have provided several recommendations for reducing CRKP incidence, including active surveillance, hand hygiene (HH), contact precautions (CP), and terminal disinfection. Various studies have been conducted to confirm the effectiveness of different IPC interventions for decreasing the CRKP incidence rate (9–14). Our previous study also showed that comprehensive IPC interventions, including de-escalation and targeted bundles, significantly reduced the incidence of CRKP colonization/infection (4). However, due to heterogeneity in study design, IPC interventions, and the definition of CRKP colonization/infection across various published studies, whether these interventions could be extrapolated to other settings remains under debate. We found a trend toward increasing cases of CRKP colonization at ICU admission during our previous study, which has remained a significant trend in recent years. The increasing incidence of CRKP detection at admission makes IPC interventions more challenging and further emphasizes the importance of pre-emptive efforts to prevent dissemination.

To extrapolate our previous IPC interventions to a different ICU setting and validate whether our interventions could effectively prevent CRKP colonization at admission, we conducted a prospective study in the newly opened ICU of a teaching hospital in Shanghai.

## Methods

### Setting and Study Design

We employed a quasi-experimental, historical control study design. The Department of Critical Care of Ruijin Hospital, which is affiliated with Shanghai Jiao Tong University School of Medicine, has two wards. One ward was newly opened in 2017. Both wards are general intensive care settings that admit patients with surgical or medical etiologies. This study was conducted in the new ICU ward, named ICU Ward Two. This ward consists of 17 beds, including seven single rooms, two triple rooms, and one quad room. Approximately 200–250 patients are admitted to the ICU annually.

ICU Ward Two was a newly opened ward, lacking any baseline incidence for comparison as a control period. Therefore, we selected patients admitted to ICU Ward One between January 2013 and June 2013 as a historical control group for the following reasons. First, we began our IPC interventions in ICU Ward One in July 2013. Between January 2013 and June 2013, only standard IPC measures were performed. Second, ICU Ward One and ICU Ward Two belong to the same Department of Critical Care Medicine, ensuring that both wards are equipped with the same IPC approach and infrastructure. Third, the compositions of the ICU staff, including doctors and nurses, are similar between the two wards, and all ICU staff attend routine IPC training courses. Fourth, critically ill patients were randomly admitted to the two wards, and the characteristics of patients, including disease severity, etiology, and age, did not differ significantly between the baseline and intervention period (Supplementary Table 1). Therefore, we used the data for patients admitted to ICU Ward One between January 2013 and June 2013 as a historical control group.

### Infection Prevention Control Interventions

For the historical control group, CP, HH, ICU staff education, routine disinfection, and sterilization were performed. Regular microbial culture surveillance at a frequency of twice a week was conducted to monitor CRKP colonization/infection incidence.

For the intervention group, multimodal IPC intervention bundles were implemented for all patients admitted to ICU Ward Two (Table 1). The details of the IPC intervention bundles can be found in our previous study (4), and no changes were made to the IPC interventions implemented during the study period.

**Table 1:** Implementation of infection prevention and control (IPC) measures during the baseline and intervention period.

**IPC measures**	**Baseline**	**Intervention**
**De-escalation interventions**		
Active surveillance cultures		X
Contact precautions and hand hygiene	X	X
Disinfection and sterilization	X	X
Department staff education	X	X
**Pre-emptive interventions**		
Contact precautions of shared equipment		X
Patient isolation: single room isolation if possible		X
**And** de-escalation interventions		X
**AMR interventions**		
Patient isolation: single room isolation or cohorting		X
Cohorting of medical care		X
Enhanced external medical staff education		X
Enhanced terminal room disinfection		X
**And** pre-emptive interventions		X
**Targeted catheter-related infection prevention bundles**		
Intravascular catheter-related infection		X
Ventilation associated pneumonia		X
Catheter-associated urinary tract infection		X

*AMR, antimicrobial resistance*.

(1) Pre-emptive prevention was applied if the patient was identified as a high risk for carrying CRKP, but no evidence of infection was found at admission. Risk factors for CRKP colonization/infection at admission included a known history of CRKP colonization/infection, transfer from a clinical department with a high risk of CRKP colonization (see Definition), or indwelling catheters, including central-line catheters, endotracheal tubes, urine catheters, or drainage catheters. The pre-emptive IPC interventions included: (i) patient isolation, including the use of a single room if possible; (ii) CP, such as HH before and after patient care and wearing gowns and gloves before entering the potentially contaminated bed unit; (iii) active surveillance cultures (ASCs) to detect pathogen colonization/infection were performed twice a week, including oral-pharyngeal swab, sputum, urine, and drainage cultures; and (iv) disinfection and sterilization were performed by environmental cleaning with sodium hypochlorite and ultraviolet (UV) light.

(2) AMR prevention was implemented in patients with confirmed colonization/infection with CRKP, as follows: (i) Patient isolation, including the use of a single room or cohorting multiple CRKP-positive patients in a triple or quad room and the management of CRKP-positive patients by dedicated nurses and physicians; (ii) CP, such as HH before and after patient care and wearing gowns and gloves before entering the potentially contaminated bed unit. Medical equipment was provided exclusively, if possible, and shared medical equipment was provided to non-CRKP patients prior to CRKP carriers; (iii) ASCs to monitor pathogen colonization/infection were performed twice a week, including oral-pharyngeal swab, sputum, urine, and drainage cultures; and (iv) disinfection and sterilization were performed by environmental cleaning with sodium hypochlorite and UV light or hydrogen peroxide vapor.

(3) De-escalation of prevention measures was prescribed in one of the following situations: (i) no evidence is found to suggest CRKP colonization/infection after two consecutive ASCs collected from multiple sites, but the patient requires various indwelling catheters; (ii) a patient with confirmed CRKP colonization/infection has two consecutive ASCs negative for CRKP over at least 1 week after 1 month of AMR prevention. The de-escalation of prevention measures included: (i) CP, such as HH before and after patient care and wearing gowns and gloves before entering a potentially contaminated bed unit; (ii) ASCs for pathogen colonization/infection were performed twice per week, including oral-pharyngeal swab and sputum, urine, and drainage cultures; and (iii) disinfection and sterilization were performed by environmental cleaning with sodium hypochlorite and UV light.

All patients received targeted bundle interventions against central-line-associated bloodstream infection (CLABSI), ventilator-associated pneumonia (VAP), and catheter-associated urinary tract infection (CAUTI). The targeted bundle to prevent CLABS included: (i) education, training, and staffing measures; (ii) the selection of catheters, sites, and catheter dressing; (iii) aseptic technique and maximal sterile barrier precautions; (iv) catheter maintenance; (v) the replacement and removal of catheters; (vi) appropriate central-vein catheter use; and (vii) surveillance for CLABSI (15, 16). The targeted bundle to prevent CAUTI included: (i) education, training, and staffing measures; (ii) proper techniques for urinary catheter insertion; (iii) proper techniques for urinary catheter maintenance; (iv) appropriate urinary catheter use; and (v) surveillance for CAUTI (17). The targeted bundle to prevent VAP included: (i) education, training, and staffing measures; (ii) appropriate ventilator use; (iii) minimized sedation; (iv) the maintenance and improvement of physical conditioning; (v) optimized airway management; (vi) elevating the head of the bed; (vii) maintaining ventilator circuits; and (viii) surveillance for VAP (18, 19).

### Bacterial Identification and Antimicrobial Susceptibility Testing

All ASCs and additional clinical cultures obtained at the clinician's discretion were sent to the Department of Clinical Microbiology for conventional testing. All bacterial isolates were identified using a Vitek 2 Compact System (bioMérieux, France), and antimicrobial susceptibility tests were performed using the Vitek 2 system and disk-diffusion assays. The susceptibility breakpoints and minimum inhibitory concentration (MIC) values were interpreted according to the Clinical and Laboratory Standards Institute guidelines (20). *Escherichia coli* ATCC 25922 was used as the quality-control strain.

CRKP was defined as KP that tested as resistant to any carbapenems (i.e., MIC ≥4 μg/mL for doripenem, meropenem, or imipenem; odds ratio ≥ 2 μg/mL for ertapenem) (21). All analyses were based on the first culture that tested as CRKP-positive during each patient's index hospitalization.

### Definition

CRKP was defined as any KP strain not susceptible to imipenem, meropenem, or ertapenem. CRKP was classified as colonization/infection at admission when detected before ICU admission or within 3 days after ICU admission. ICU-acquired colonization/infection of CRKP was confirmed when pathogens were not present at the time of admission but were detected after ICU admission for > 3 days (22). Clinical departments with a high risk of CRKP colonization were defined as those with a high incidence of CRKP. CLABSI, VAP, and CAUTI were defined according to the Centers for Disease Control and Prevention guidelines (23). Each CRKP-positive case was evaluated independently by two physicians from our specific infectious disease expert team.

The incidence of CRKP isolates detected in cultures was measured and standardized as the number of cases per 1,000 ICU patient-days. The incidence of ICU-acquired CRKP colonization/infections (cases per 1,000 ICU patient-days) was calculated (10, 24). The incidence of CRKP at admission (cases per 100 ICU patient-admission) was calculated to evaluate the prevalence of patients introducing CRKP into the ICU. The monthly, annual, and periodic incidence of CRKP positivity were calculated.

The primary outcome was the monthly incidence rate of ICU-acquired CRKP (cases per 1,000 ICU patient-days) during the research period. Subsequently, the secondary outcomes included the incidence of CRKP at admission, the incidence of catheter-related infections from various sites (cases per 1,000 catheter-days), and IPC intervention compliance.

### Data Collection

Clinical characteristics and microbiological information were collected from the Hospital Information System by two experienced ICU medical doctors. Information regarding patient demographics, comorbidities, and the probable source of infection was collected. The general health status and prior healthcare exposure of patients were assessed at the time of culture collection. Additionally, the severity of illness at the time of culture collection was assessed using the Acute Physiology and Chronic Health Evaluation (APACHE II) and Sequential Organ Failure Assessment score (SOFA score).

### Assessment of Compliance With Multimodal IPC Interventions

To estimate the effects of various multimodal IPC interventions on improving healthcare workers' compliance, we counted the consumptions of sterile and non-sterile gloves from the hospital data. Baseline compliance and compliance after the intervention implementation were assessed using the same methodology. Additionally, correct disinfection behavior and the sequence of disinfection were monitored, and the compliance with terminal room disinfection procedures was evaluated by reviewing monitoring videos quarterly during the intervention period. The data collection was performed as a prevalence point and included all health care workers (HCWs), such as physicians, nurses, respiratory therapists, and technical assistants.

### Statistical Analysis

The results were analyzed using a commercially available statistical software package (SPSS 25.0; IBM, Armonk, NY, USA). Discrete variables are summarized as the frequency (%) and continuous variables as the mean and standard deviation (SD) or the median and interquartile range (IQR). Segmented linear regression was performed to evaluate gradual changes in the outcome parameters during the segment. To detect significant differences between groups, we used the Chi-square test or Fisher's exact test for categorical variables and the two-tailed *t*-test and the Mann–Whitney *U* test for continuous variables, as appropriate. Significance was established at *p* < 0.05. All reported p-values are two-tailed.

## Results

### Demographic Data of Patients With CRKP Colonization/Infection

CRKP isolates were identified in 72 patients in the ICU during the study period; 40 patients were identified with ICU-acquired CRKP, including 21 during the baseline period and 19 during the intervention period. Patient data, such as general characteristics, CRKP culture sites, exposure to therapy before CRKP colonization/infection, and outcomes, are described in Table 2. The digestive barrier destruction before CRKP colonization/infection and the proportion of patients with CRKP isolated from the abdominal cavity were higher during the baseline period than during the intervention period. However, the proportion of patients with CRKP isolated from the respiratory tract was higher during the intervention period than during the baseline period. All other patient characteristics during the ICU stay were similar between the two periods.

**Table 2:** Clinical characteristics of the patients with ICU-acquired carbapenem-resistant *Klebsiella pneumoniae* (CRKP).

**Variable**	**Baseline (*n* = 21)**	**Intervention (*n* = 19)**	***P***
**General characteristics**			
Age, mean ± SD	66.0 ± 15.3	62.9 ± 15.6	0.536
Male (%)	16 (76.2)	14 (73.7)	1
APACHE II score (IQR)	16 (11.5–21)	18 (14.5–23.5)	0.314
SOFA score (IQR)	4.5 (2.5–7)	5 (2.5–8.5)	0.375
Duration (IQR)	14 (8–30)	11 (7.5–23)	0.489
**Culture site**			
Respiratory tract (%)	4 (19.0)	10 (52.7)	0.026
Abdominal cavity (%)	15 (71.4)	2 (10.5)	0.000
Urine (%)	1 (4.8)	3 (15.8)	0.331
Bloodstream (%)	1 (4.8)	2 (10.5)	0.596
Skin and soft tissue (%)	0	2 (10.5)	0.219
**Exposure of therapy before CRKP colonization/infection**			
Carbapenem (%)	17 (81.0)	14 (73.7)	0.712
Central venous catheter (%)	18 (85.7)	15 (78.9)	0.689
Urinary catheter (%)	20 (95.2)	17 (89.5)	0.596
Mechanical ventilation (%)	15 (71.4)	11 (57.9)	0.370
Artificial airway (%)	15 (71.4)	10 (52.6)	0.220
Digestive barrier destruction (%)	18 (85.7)	7 (36.8)	0.001
**Outcome**			
Infection (%)	11 (52.4)	5 (26.3)	0.093
LOS in ICU (IQR)	57 (19–83)	28 (17.5–69)	0.498
28-day mortality (%)	2 (9.5)	3 (15.8)	0.654
In-ICU mortality (%)	4 (19.0)	4 (21.1)	1
Hospital mortality (%)	5 (23.8)	5 (26.3)	1

*ICU, intensive care unit; SD, standard deviation: APACHE, Acute Physiology and Chronic Health Evaluation; SOFA, Sequential Organ Failure Assessment; IQR, interquartile range; LOS, length of stay*.

### Incidence of CRKP Colonization/Infection During the Baseline and Intervention Periods

**Figure 1:**
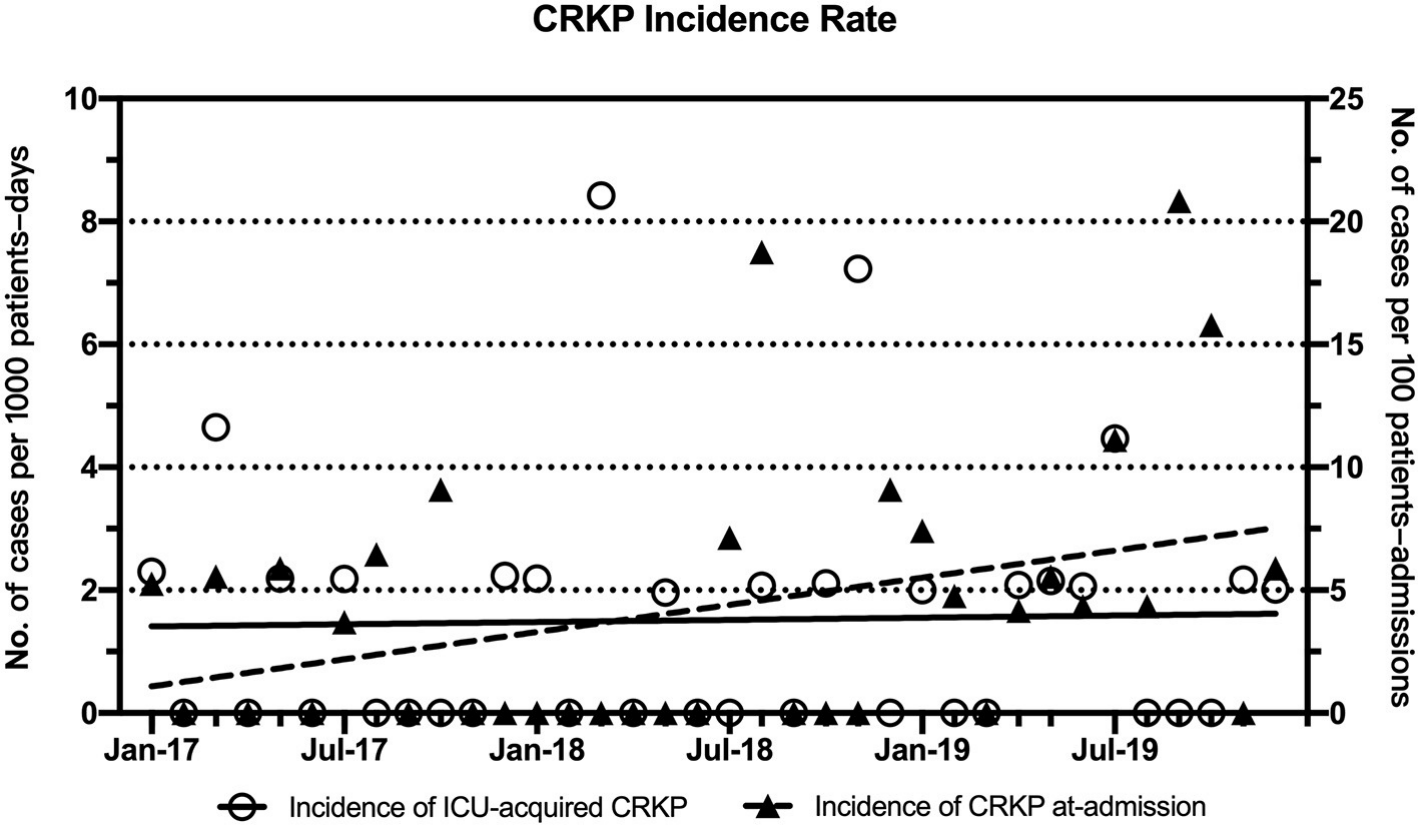
Trends in the monthly incidence rates (cases per 1,000 ICU patient-days) for ICU-acquired CRKP and CRKP at admission (cases per 100 ICU patient-admissions) during the intervention period. Linear fitting for ICU-acquired CRKP (*y* = 0.0059x + 1.404) is shown as a black solid line, and linear fitting for CRKP at admission (*y* = 0.1842x + 0.901, *R*^2^ = 0.1252, *p* = 0.03) is shown as a dotted line.

The periodic and annual incidence of CRKP colonization/infections, including the incidence of ICU-acquired CRKP and CRKP at admission, are shown in Tables 3, 4. Although no significant difference in the periodic incidence of CRKP at admission was observed between the baseline and intervention periods (1.14 vs. 4.70 cases per 100 patient-admissions, respectively, *p* = 0.175), the slope of the monthly incidence of CRKP at admission (Figure 1) showed an increasing trend during the intervention period (*p* = 0.03). Compared with the baseline period, the annual incidence of CRKP at admission in 2019 also showed a significant change (0.00 [0.00–1.92] vs. 5.28 [2.18–10.32], respectively, *p* = 0.044).

**Table 3:** Epidemiology of patients with culture isolates identified as carbapenem-resistant *Klebsiella pneumoniae* (CRKP).

	**Baseline**	**Intervention**	***p***
Total patients (*N*)	88	660	–
Patient-days (day)	2,060	16,426	–
Imported CRKP carriers (*N*)	1	31	–
ICU-acquired CRKP patients (*N*)	21	19	–
Incidence of CRKP at admission (cases per 100 patient-admission)	1.14	4.70	0.175
Incidence of ICU-acquired CRKP (cases per 1,000 patient-days)	10.19	1.16	0.002

*ICU, intensive care unit*.

**Table 4:** Incidence of ICU-acquired CRKP and CRKP at admission during the baseline and intervention periods.

**Period**	**Incidence of CRKP at admission**				**Incidence of ICU-acquired CRKP**			
	**Cases per 100 patient-admission**				**Cases per 1,000 patient-days**			
	**Mean**	**Median**	***p***^**a**^	***p***^**b**^	**Mean**	**Median**	***p***^**c**^	***p***^**d**^
Baseline	1.28 ± 3.14	0.00 (0.00–1.92)	–	–	9.88 ± 6.44	10.08 (4.43–16.43)	–	–
2017	3.15 ± 3.53	1.85 (0.00–6.01)	0.263	–	0.96 ± 1.54	0.00 (0.00–2.27)	0.005	–
2018	2.19 ± 4.00	0.00 (0.00–3.57)	0.653	0.445	1.27 ± 1.53	0.98 (0.00–2.11)	0.009	0.747
2019	6.9 9 ±6.46	5.28 (2.18–10.32)	0.044	0.146	1.26 ± 1.46	1.00 (0.00–2.17)	0.009	0.797

*p^a^, compared with the baseline period; p^b^, compared with 2017; p^c^, compared with the baseline period; p^d^, compared with 2017; CRKP, carbapenem-resistant Klebsiella pneumoniae; ICU, intensive care unit*.

The periodic incidence of ICU-acquired CRKP was lower during the intervention period (1.16 per 1,000 patient-days) than during the baseline period (10.19 per 1,000 patient-days, *p* = 0.002), as shown in Table 3. After the implementation of the multimodal IPC intervention, the annual incidence of ICU-acquired CRKP stabilized and decreased significantly in 2017 (0 per 1,000 patient-days, IQR, 0-2.27), 2018 (0.98 per 1,000 patient-days, IQR, 0–2.11), and 2019 (1.00 per 1,000 patient-days IQR, 0–2.17) compared with the baseline period (Table 4). No significant differences in the ICU-acquired CRKP incidences were observed among the intervention periods (*p* = 0.747 of a comparison between 2017 and 2018, and *p* = 0.797 of a comparison between 2017 and 2019).

### Identification of CRKP Colonization/Infection Sources at Admission

The sources of CRKP colonization/infections identified at admission were recognized in the intervention (Table 5). The incidence of CRKP at admission was high among patients transferred from other medical facilities (18.0%, 9/50) and from the Emergency Department (6.9%, 4/58). CRKP incidence was observed at a low level among patients transferred from surgical departments (3.4%, 16/470). Remarkably, patients who underwent pancreatic surgery (10.8%, 14/129) were more likely to be CRKP carriers at admission.

**Table 5:** The sources of CRKP during the intervention period.

	**Intervention**	**2017**	**2018**	**2019**	***p***
Hospital-acquired	31 (4.7%)	7 (3.0%)	6 (3.3%)	18 (7.2%)	0.056
Other facility	9 (18.0%)	1 (5.9%)	3 (17.6%)	5 (31.3%)	0.154
Medical ward	2 (2.9%)	0	0	2 (6.3%)	0.495
Emergency department	4 (6.9%)	1 (4.5%)	0	3 (11.1%)	0.645
ICU	0	0	0	0	–
Surgical ward	16 (3.4%)	5 (2.9%)	3 (2.2%)	8 (4.8%)	0.440
Pancreatic surgery	14 (10.8%)	4 (10.8%)	2 (4.9%)	8 (16.0%)	0.215

*P, compared between intervention periods*.

*CRKP, carbapenem-resistant Klebsiella pneumoniae; ICU, intensive care unit*.

### Efficacy of Catheter-Targeted ICP Intervention Bundles

As part of the comprehensive IPC interventions, the incidence of ICU-acquired catheter-related infections caused by CRKP (cases per 1,000 catheter-days) was evaluated (Table 6). ICU-acquired CRKP-positive CLABSI and VAP incidence decreased from 2.54 to 0.96 per 1,000 central-line-days and from 2.84 to 0.00 infections per 1,000 ventilator-days, respectively.

**Table 6:** Incidence of ICU-acquired catheter-related infections caused by CRKP (cases per 1,000 catheter-days) from different infection sites in the study period.

	**Baseline**	**Intervention**	**2017**	**2018**	**2019**	***p***
Central-line-associated bloodstream infection (cases per 1,000 central-line-days)	2.54	0.96	1.07	1.14	0.63	0.08
Ventilator-associated pneumonia (cases per 1,000 ventilator-days)	2.84	0.00	0.00	0.00	0.00	–
Catheter-associated urinary tract infection (cases per 1,000 catheter-days)	0.00	0.00	0.00	0.00	0.00	–

*P, compared between baseline and intervention period; CRKP, carbapenem-resistant Klebsiella pneumonia*.

### Compliance With Multimodal IPC Interventions

Compliance with CP, including the use of both sterile and non-sterile gloves, increased from 517 and 2,842 pairs per month before the intervention to 581 and 11,047 pairs per month after the intervention, respectively. Compliance with terminal room disinfection improved during the intervention period, from 64.5 to 94.3%, including a total of 1,127 observations (*p* < 00.001). The collection of environmental surface cultures was implanted after terminal room disinfection, and all culture samples were negative for CRKP.

## Discussion

In this study, we extrapolated and validated whether our comprehensive IPC interventions could be implemented to effectively reduce and maintain a low incidence of ICU-acquired CRKP colonization/infection in an epidemic region and the incidence of CRKP-associated ICU-acquired CLABSI and VAP.

CRKP has become a global health problem and is listed in the critical priority category of the 2019 WHO global priority pathogens list for the research and development of new antibiotics. The incidence of ICU-acquired CRKP colonization/infection varies between 3.7 to 8.1 cases per 1000 patient-days, according to different studies worldwide (25, 26). Data from the China Antimicrobial Surveillance Network (CHINET) showed that the prevalence of CRKP has been markedly increasing in China, from 2.6% in 2005 to 28.6% in 2018. CRKP has spread extensively in Shanghai at the province level, reaching an incidence of 47.7% in 2018 (27, 28). We found an increasing trend in the number of CRKP isolation cases in our hospital. The annual incidence of CRKP isolation at Ruijin Hospital increased from 0.29 per 1,000 patient-days in 2017 to 0.36 per 1,000 patient-days in 2019. Across all of the ICUs (including surgical, medical, emergency, and general ICU wards) in Ruijin Hospital, the annual incidence of CRKP isolation was 6.30, 5.12, and 5.40 per 1,000 patient-days for 2017, 2018, and 2019, respectively. The overall annual incidence of CRKP in ICU Ward Two was 2.33, 2.31, and 4.41 per 1,000 patient-days for 2017, 2018, and 2019, respectively. The high density of CRKP in healthcare facilities has resulted in more patients being colonized with CRKP at admission, which challenges the IPC interventions. We found an increasing trend in the number of patients with CRKP colonized at ICU admission in our previous study, and this trend has remained significant in recent years. The increasing incidence of CRKP at admission makes IPC interventions more challenging, emphasizing the importance of pre-emptively preventing dissemination.

Studies have shown that several pathways could cause horizontal nosocomial transmission of CRE, including healthcare worker's hands, shared equipment, and the healthcare environment. Therefore, infection control bundles aim to halt these routes of CRKP transfer by acting at different levels of the transmission pathways (29). In recent decades, various studies have reported the successful containment of nosocomial CRKP outbreaks in epidemic regions through the implementation of IPC bundles consisting of diverse interventions. A meta-analysis showed that the most effective interventions for the prevention of multidrug-resistant gram-negative bacteria (MDR-GNB) acquisition in adult ICUs were comprised of a 4-component strategy, including standard care, an antimicrobial stewardship program, environmental cleaning protocols, and source control, which were highly successful at reducing infections compared with standard care alone (rate ratio [RR]: 0.05; 95%CI: 0.01–0.38) (30). In an ICU in Greece, the incidence of CRKP infections decreased significantly from a median of 19.6 per 1000 patient-days to 8.1 per 1,000 patient-days after the implementation of IPC bundles (*p* = 0.001) (25).

AMR strategies, including standard care, an antimicrobial stewardship program, environmental cleaning, and source control, were protocolized in our hospital. However, the overall and ICU-acquired incidence of CRKP continued to increase in the hospital and ICU settings. We began to control the cross-transmission of CRKP, starting in 2013, using a comprehensive IPC intervention program combined with targeted catheter-related IPC bundles (4). This strategy differed from our hospital's overall AMR strategy by implementing ASCs, pre-emptive patient isolation, infection control for shared medical equipment, and enhanced environmental cleaning. In our previous study, the incidence of ICU-acquired CRKP colonization/infections decreased following the implementation of the IPC interventions and stabilized at a low rate in ICU Ward One (2.84 cases per 1,000 patient-days, IQR: 2.80–2.89) during the follow-up period (July 2015 to June 2016). A similar multimodal IPC strategy was extrapolated and conducted in the new ward, and the incidence was 0.00 cases per 1,000 patient-days (IQR 0.00–2.15) during the intervention period (2017–2019, ICU Ward Two). A significant difference was observed in the monthly incidence rates between the follow-up period in Ward One and the intervention period in Ward Two (2.84 vs. 0.00 cases per 1,000 patient-days, *p* = 0.00005) (4). This multimodal IPC strategy was effective in Ward Two compared with the baseline group and the follow-up period in ICU Ward One. Moreover, we found the incidence of ICU-acquired CRKP remained at a steady and low level during the intervention period in ICU Ward Two, despite an increasing incidence of CRKP colonization/infection at admission, which also indicated the effectiveness of our interventions. Last but not least, the standardization of incidence evaluations by the Centers for Disease Control and Prevention also enables our results to be compared against those of other studies (10, 24). We found that the incidence of ICU-acquired CRKP colonization/infection varied between 3.7 and 8.1 cases per 1000 patient-days, according to different studies (25, 26). The persistently low incidence level observed after implementing multimodal IPC interventions in our study was meaningful when compared to other study results.

Through the use of ASCs, an increasing trend in CRKP colonization/infection incidence at ICU admission was observed in our study, especially in 2019. Genomic analysis revealed that transmission rates at the facility level are associated with facility-level CRKP prevalence, and higher intrafacility transmission rates drove facility-level variations in the levels of CRKP prevalence (Spearman's *R* = 0.75, *p* = 0.012) (31). The high influx of CRKP carriers from other facilities and departments may result in high colonization pressure. High colonization pressure was associated with the spread of antibiotic-resistant bacteria in several studies (32, 33). Reports suggest that the relative burden of asymptomatic CRE carriage may be high (34), and future studies remain essential for validating these results using combinations of clinical and ASCs.

Our data also indicated that patients who transferred from other healthcare facilities have the highest CRKP incidence at admission. Additionally, the emergency department and pancreatic surgery were associated with a high incidence of CRKP at admission among patients transferred to the ICU. Whole-genome sequencing indicated that high regional CRKP burdens were due to a small number of regional introductions, with subsequent regional proliferation occurring via patient transfers among healthcare facilities (31). The extensive sharing of complex patients among healthcare facilities within a region can facilitate pathogen spread in the absence of focused preventative efforts, suggesting that effective CRE control requires a coordinated regional effort across all acute and long-term healthcare facilities (14). After an active regional infection control intervention was applied, the overall incidence of multidrug-resistant enteric bacteria decreased from 2.2 to 0.5% (*p* < 00.001) across all healthcare facilities (35). A recent model predicted that a coordinated response to prevent the spread of CRE across interconnected healthcare facilities would result in a 55% reduction in CRE acquisitions over 15 years (36). Further investigation of this potential outcome that targets high-incidence departments remains necessary.

High-quality HH and CP may reduce bacterial transmission by healthcare staff (37, 38). In response, international public health agencies, including the WHO, have recommended the enforcement of HH and CP as cornerstones of IPC interventions. The increased compliance with glove-wearing showed the reliable implementation of HH and CP. Compared with the reference group, the incidence of multidrug-resistant organisms among exposed patients was significantly lower (relative risk: 0.70, 95%CI: 0.50–0.98; *p* = 0.036) following the addition of UV light to standard cleaning strategies (39). In the present study, in addition to standard disinfection strategies, UV light and hydrogen peroxide vapor were applied during terminal room disinfection. Compliance monitoring ensured the implementation of enhanced terminal room disinfection protocols, which reduced CPKP-spread through contaminated environmental surfaces.

In parallel with the reduced incidence of ICU-acquired CRKP colonization/infection, a marked reduction in catheter-related CRKP infection was observed in the intervention period. The overall incidence of CLABSI, VAP, and CAUTI caused by any organisms is listed in Supplementary Table 2. The incidence of CLABSI caused by CRKP decreased during the intervention period, whereas the annual incidence of CLABSI and VAP caused by all organisms experienced fluctuations during the same period (Supplementary Table 2). An increasing trend was observed in the annual incidence of CAUTI caused by all organisms (2.47, 3.93, and 7.62 cases per 1,000 catheter-days for 2017, 2018, and 2019, respectively). An increase in annual urinary catheter utilization was observed (0.39, 0.50, and 0.53, measured as catheter-days divided by patient-days, for 2017, 2018, and 2019, respectively). A meta-analysis showed the implementation of central-line bundles in ICU decreased the incidence of infections significantly from a median of 6.4 per 1,000 catheter-days (IQR 3.8–10.9) to 2.5 per 1,000 catheter-days (IQR 1.4–4.8) (40). A multimodal CAUTI intervention strategy, including early urinary catheter removal, resulted in measurable decreases in both urinary catheter utilization (0.78–0.70) and CAUTI rates (5.1–2.0 cases per 1,000 catheter-days) in the ICU (41). The early removal of urinary catheters represents an effective strategy for CAUTI prevention. Healthcare improvement programs have been shown to be effective for preventing the development of complications related to intravascular catheters, especially when combined with local compliance (15). When an infection control infrastructure aimed at CRE is established and well-operated, numerous IPC successes can be achieved, including the containment of a CRE outbreak, a marked reduction in the incidence of CLABSI in ICUs, and reduced overall ICU-acquired bloodstream infections, including the successful containment of nosocomial infections caused by other multidrug-resistant pathogens (42). The fluctuating incidence observed for catheter-related infections may be due to changes in compliance with the bundles. Strategies for early catheter removal and compliance with various interventions should be assessed in further studies.

Our study had a few limitations. First, the study used a historical control design. The results of our study might be more robust if we compared our intervention group with another group at the same time or with a baseline in the same setting. We plan to conduct a prospective, randomized, multicenter study to further extrapolate and validate the efficacy of our comprehensive IPC interventions. Second, the anal swab was not included in the multisite ASC in this study, which might result in an underestimation of the incidence of CRKP colonization. We plan to include anal swabs in our daily practice and in future studies.

## Conclusion

In epidemic settings with a high incidence of CRKP, multimodal IPC intervention strategies and the consistent monitoring of compliance are effective and practical methods for preventing CRKP dissemination in ICUs. However, due to the historical control design of our study, the efficacy of our intervention strategies continues to require further validation in a prospective, multicenter, parallel study.

## Data Availability Statement

The raw data supporting the conclusions of this article will be made available by the authors, without undue reservation.

## Ethics Statement

Multimodal IPC measures have become routine processes in our ward; therefore, the consent form was exempted in this study. The study protocol on collection and analysis of patients' clinical and microbiological information was approved by the Institutional Ethics Committees of Ruijin Hospital Affiliated to Shanghai Jiao Tong University School of Medicine (Approval No: 2019–1-3).

## Author Contributions

HQ, JL, and RT designed the study. YDa, TM, and XW analyzed the data. BT, FW, YDu, and YQ performed the surveillance. YDa and RT wrote the manuscript. All authors read and approved the final manuscript.

## Conflict of Interest

The authors declare that the research was conducted in the absence of any commercial or financial relationships that could be construed as a potential conflict of interest.
