# Supplementary material for: Validation and Extrapolation of a Multimodal Infection Prevention and Control Intervention on Carbapenem-Resistant Klebsiella pneumoniae in an Epidemic Region: A Historical Control Quasi-Experimental Study
Source: Front Med (Lausanne). 2021 Jul 7;8:692813. doi: 10.3389/fmed.2021.692813 (PMC8292674; doi:10.3389/fmed.2021.692813)
Supplement: Supplementary file 1 [file Table_1.DOCX]

**Supplementary Table S1.** Clinical characteristics of the patients in the study periods

|  | **Baseline** | **Intervention** | **p-value** |
| --- | --- | --- | --- |
| Total patients (N) | 88 | 660 |  |
| Male (%) | 60 (68.2) | 413 (62.6) | 0.277 |
| Age, mean ± SD | 62.5 ± 17.2 | 64.7 ± 17.9 | 0.202 |
| APACHE II score (IQR) | 13 (11–17) | 14 (10–20) | 0.409 |
| ICU duration (IQR) | 8 (3–20) | 7 (4–17) | 0.490 |
| ICU mortality (%) | 12 (13.6) | 70 (10.6) | 0.466 |

SD: standard deviation; APACHE: Acute Physiology and Chronic Health Evaluation; IQR: interquartile range; ICU: intensive care unit

**Supplementary Table S2.** Incidence of ICU-acquired, catheter-related infections caused by all organisms and by CRKP from different infection sites in the study period.

|  | **Incidence (cases per 1000 catheter-days, all organisms)** | | | **Incidence (cases per 1000 catheter-days, CRKP)** | | |
| --- | --- | --- | --- | --- | --- | --- |
| **Period** | **CLABSI** | **CAUTI** | **VAP** | **CLABSI** | **CAUTI** | **VAP** |
| 2017 | 1.07 | 2.47 | 2.35 | 1.07 | 0.00 | 0.00 |
| 2018 | 3.43 | 3.93 | 6.30 | 1.14 | 0.00 | 0.00 |
| 2019 | 0.63 | 7.62 | 2.82 | 0.63 | 0.00 | 0.00 |

CKRP: carbapenem-resistant *Klebsiella pneumoniae*; CLABSI: central line-associated bloodstream infection; CAUTI: catheter-associated urinary tract infections; VAP: ventilator-associated pneumonia
